# Potential Animal Reservoir of *Mycobacterium ulcerans*: A Systematic Review

**DOI:** 10.3390/tropicalmed3020056

**Published:** 2018-05-30

**Authors:** Avishek Singh, William John Hannan McBride, Brenda Govan, Mark Pearson

**Affiliations:** 1 Cairns Clinical School, College of Medicine and Dentistry, James Cook University, Cairns City, QLD 4870, Australia; john.mcbride@theiddoctor.com; 2 College of Public Health, Medical & Vet Sciences, James Cook University, Townsville, QLD 4811, Australia; brenda.govan@jcu.edu.au; 3 Australian Institute of Tropical Health & Medicine, James Cook University, Smithfield, QLD 4878, Australia; mark.pearson@jcu.edu.au

**Keywords:** *Mycobacterium ulcerans*, animal reservoir, transmission

## Abstract

*Mycobacterium ulcerans* is the causative agent of Buruli ulcer, also known in Australia as Daintree ulcer or Bairnsdale ulcer. This destructive skin disease is characterized by extensive and painless necrosis of the skin and soft tissue with the formation of large ulcers, commonly on the leg or arm. To date, 33 countries with tropical, subtropical and temperate climates in Africa, the Americas, Asia and the Western Pacific have reported cases of Buruli ulcer. The disease is rarely fatal, although it may lead to permanent disability and/or disfigurement if not treated appropriately or in time. It is the third most common mycobacterial infection in the world after tuberculosis and leprosy. The precise mode of transmission of *M. ulcerans* is yet to be elucidated. Nevertheless, it is possible that the mode of transmission varies with different geographical areas and epidemiological settings. The knowledge about the possible routes of transmission and potential animal reservoirs of *M. ulcerans* is poorly understood and still remains patchy. Infectious diseases arise from the interaction of agent, host and environment. The majority of emerging or remerging infectious disease in human populations is spread by animals: either wildlife, livestock or pets. Animals may act as hosts or reservoirs and subsequently spread the organism to the environment or directly to the human population. The reservoirs may or may not be the direct source of infection for the hosts; however, they play a major role in maintenance of the organism in the environment, and in the mode of transmission. This remains valid for *M. ulcerans*. Possums have been suggested as one of the reservoir of *M. ulcerans* in south-eastern Australia, where possums ingest *M. ulcerans* from the environment, amplify them and shed the organism through their faeces. We conducted a systematic review with selected key words on PubMed and INFORMIT databases to aggregate available published data on animal reservoirs of *M. ulcerans* around the world. After certain inclusion and exclusion criteria were implemented, a total of 17 studies was included in the review. A variety of animals around the world e.g., rodents, shrews, possums (ringtail and brushtail), horses, dogs, alpacas, koalas and Indian flap-shelled turtles have been recorded as being infected with *M. ulcerans*. The majority of studies included in this review identified animal reservoirs as predisposing to the emergence and reemergence of *M. ulcerans* infection. Taken together, from the selected studies in this systematic review, it is clear that exotic wildlife and native mammals play a significant role as reservoirs for *M. ulcerans.*

## 1. Introduction

Sir Albert Cook, a British missionary doctor appointed at the Mengo Hospital in Kampala, Uganda, first noted the skin ulcer caused by *Mycobacterium ulcerans* in 1896. Later, in the late 1930s, two general practitioners, Drs. J. R. Searl and D. G. Alsop, working in rural Victoria, Australia, noticed a group of cases of mysterious skin ulcers around the town of Bairnsdale [1]. The cases were not published in the literature at the time and the causative organism was not identified or characterized. Professor Peter MacCallum and his colleagues first provided the detailed description of the disease in 1948, using presentation data of six patients in the Bairnsdale district, near Melbourne. They were the first to isolate *M. ulcerans* as the causative organism of the mysterious skin ulcer [2]. The first large cluster of *M. ulcerans* infection was identified in the Buruli County of Uganda (now called Nakasongola District) in the 1960s and the disease was termed ‘Buruli ulcer’ (BU) thereafter [3].

There have been several known outbreaks of Buruli ulcer around the world and each outbreak has its own unique characteristics in terms of epidemiology and the animals reported to be involved in transmission [4,5]. The World Health Organization (WHO) has classified BU as a neglected tropical disease [6]. Presently, BU has been reported (but not always microbiologically confirmed) in more than 30 countries spread over Africa, the Americas, Asia, and Oceania [7]. Australia is the only developed country with significant local transmission of BU, with foci of infection in tropical Far North Queensland [8,9], the Capricorn Coast region of central Queensland [10], the Northern Territory [11] and temperate coastal Victoria [10]. Non-human cases of *M. ulcerans* are prevalent in Australia only, where several cases of BU have been described in both native wildlife and domestic mammal species such as koalas (*Phascolarctos cinereus*) [12,13], common ringtail possums (*Pseudocheirus peregrinus*) [14,15], a mountain brushtail possum (*Trichosurus cunninghami*) [5,14,15], two horses [16], an alpaca [17], four dogs [18] and a cat [19]. Recent research in Victoria, Australia, has suggested the transmission of infection by mosquitoes, and possums with chronic BU as an important environmental reservoir of *M. ulcerans* in Victoria [14].

## 2. Materials and Methods

The PRISMA guidelines developed by the Centre for Review Dissemination (CRD) were used as the methodology for the systematic review [20]. A review protocol was registered with PROSPERO international prospective register of systematic reviews, which can be viewed online [21]. The systematic literature review was conducted using online databases MEDLINE and INFORMIT to aggregate all the published literature. Initially, MEDLINE was used to retrieve all the scientific information concerning the research topic. INFORMIT was searched with same search strategies adopted for MEDLINE. The following key words were chosen after a series of trial searches in order to ensure an adequate number of relevant articles were reviewed: (Buruli OR ‘*Mycobacterium ulcerans*’) AND (Host OR Vector OR Reservoir OR Animal), accessed on 6 May 2018. The title and abstract of each of the articles were initially scanned to ensure that the included articles met the aim and scope of the systematic review. Articles that were deemed irrelevant to the aim of this systematic review or out of the research scope were excluded. For those articles that were not clear by the title and abstract, the full text was retrieved and further analyzed in order to determine if they met the inclusion and exclusion criteria below. The studies that reported only experimental or laboratory exposure of *M. ulcerans* in animals were excluded. The search strategy exclusively focused on potential animal reservoirs, not the vectors. The detection of the causative agent had to be confirmed by culture of bacteria and/or PCR. To be considered positive a sample needed to be confirmed either by culture of bacteria or positive for IS 2404 and reconfirmed by KR and IS 2606. Undoubtedly, PCR targeting IS 2404 is highly specific for detecting *M. ulcerans* in clinical specimen [22]. However, for detecting *M. ulcerans* from environmental samples, confirmatory PCR targeting two additional insertion sequences, IS 2606 and the ketoreductase B domain (KR), is essential to differentiate *M. ulcerans* from other environmental mycobacteria that may carry IS 2404 and other non-mycolactone-producing mycobacteria [22]. Thus, IS 2404-PCR used in conjunction with IS 2606 and KR-PCR confirms that the detected organism is *M. ulcerans.* There were no language restrictions. Risk of bias was assessed by one reviewer on the basis of independent factors such as sample size, location and nature of infection. 

## 3. Results

### 3.1. Results of the Literature Search and Method of Inclusion

**Figure 1:**
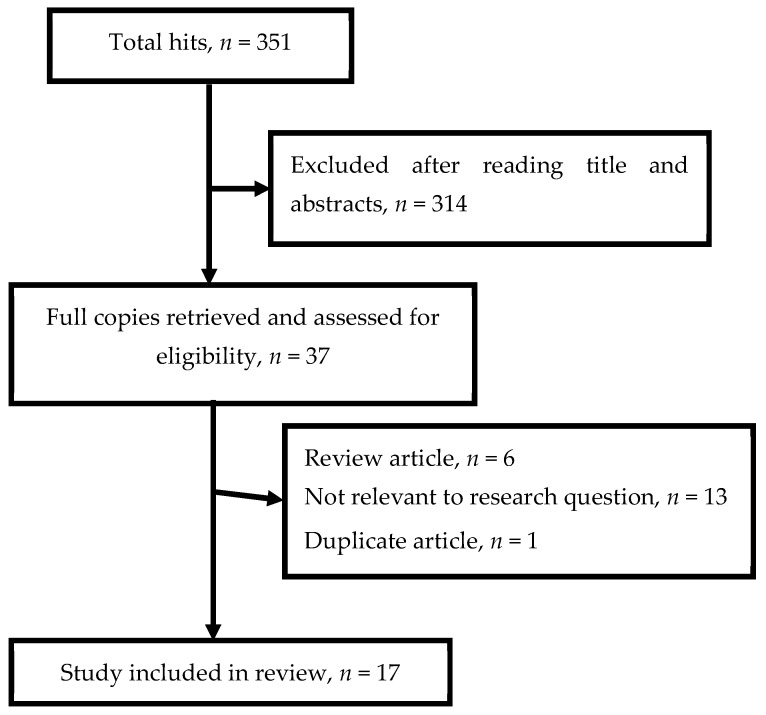
Flow chart of study selection process.

The total number of discovered articles in MEDLINE database was 351. Three hundred and fourteen articles were excluded after reading the title and abstracts as they were not relevant to the research question. Full texts of thirty-seven studies were retrieved in portable document format (PDF) for further analysis. Of these remaining 37 studies, 19 were excluded as they clearly did not meet inclusion criteria (i.e., they were review articles, focused on vectors rather than on animal reservoirs, or pertained to laboratory or experimental exposure). One additional duplicate article was excluded as well. The remaining 17 studies from the PubMed database were included for systematic review. There were no additional articles in INFORMIT that did not appear in the initial MEDLINE search results. The flow chart for study selection process is shown in Figure 1.

### 3.2. Basic Characteristics of Selected Studies

Out of the 17 included studies, ten were conducted in Australia, two in Ghana and one was conducted in each of Ivory Coast, North America, United States, Benin and Japan. The basic characteristics of selected studies for review are shown in Table 1 below.

## 4. Discussion on Possible Reservoirs and Vectors of *Mycobacterium ulcerans* by Country

This systematic review assessed the potential animal reservoir of *M. ulcerans* around the world recorded to date. This is essential for understanding the epidemiology and mode of transmission of the disease, which subsequently aids in prevention, control and elimination strategies.

### 4.1. Australia

Out of 17 studies included in this review, 10 were conducted in Australia. In Australia, the disease is more prevalent in the southeastern state of Victoria and in Far North Queensland. After the detection of *M. ulcerans* infection in four koalas in 1980 at Raymond Island, Australia [13], the entire island was searched for koalas in the following year. Thirty-six male and 51 female koalas were captured and examined. Of these, 18 out of 87 animals had skin wounds and 11 were found positive for *M. ulcerans*. Diagnosis was made on pathological and bacteriological examination; the PCR-based method used for the identification of *M. ulcerans* from clinical and environmental samples was only implemented in 1996 [30]. Non-human cases of *M. ulcerans* in Australia have been reported in marsupial species such as koalas [13], ringtail and brushtail possums [14,15,31], horses [16], alpacas [17], dogs [18] and cats [19]. A study conducted by Fyfe and colleagues between 2007–2009, at Point Lonsdale, a small coastal town south east of Melbourne, Australia, which is also endemic for BU, found that 43% of ringtail possum and 29% of brushtail possum faecal samples were positive for *M. ulcerans* DNA [14]. Only 1% of faecal samples from non-endemic area possums were positive for *M. ulcerans* DNA in this study, suggesting terrestrial mammals such as possums are potential reservoirs of *M. ulcerans* in southeast Australia. Several studies have identified possums (both ringtail and brushtail) as potential reservoirs since then [5,15]. In Australia, other than the southeastern state of Victoria, BU is also prevalent in Far North Queensland [8]. Inspired by the evidence of possums as potential reservoirs of *M. ulcerans* in Victoria, a study conducted by Roltgen and colleagues (2013) in northern Queensland, Australia, detected *M. ulcerans* DNA from two bandicoot faecal samples, suggesting the possibility that bandicoots are a potential reservoir of *M. ulcerans* in Far North Queensland [9].

### 4.2. Africa

Out of the 17 studies included in this review, four were conducted in West African countries: two in Ghana [23,25], one in the Ivory Coast [24] and one in Benin [27]. Durnez and colleagues (2006) caught 326 rodents and 222 shrews from endemic and non-endemic villages of Benin and tested for *M. ulcerans,* but no specific DNA was detected from any of their samples [27]. Despite their results, they suggested the necessity of more intensive research focusing on small mammals in Africa. Willson reported positive PCR with IS 2404 only from tadpoles and fishes from Ghana [25]. Similarly, two faecal specimens from *Thryonomys swinderianus* (agouti) were reported positive for *M. ulcerans* in a study conducted by Bi Diangoné Tian and colleagues (2014) from the Ivory Coast [24]. They suggested agouti, which are closely related to Australian possums, could be a potential reservoir of *M. ulcerans* in Africa. However, RT-PCR targeting IS 2606 was not conducted to confirm *M. ulcerans*. A faecal survey of domestic animals in rural Ghana for *M. ulcerans* conducted by Tobias and associates suggested no evidence of association between domestic animals and *M. ulcerans* in endemic and non-endemic villages in Ghana [23]. Unlike Australia, not a single study in Africa has reported the presence of *M. ulcerans*-positive DNA or cases in non-human species, suggesting that transmission dynamics may be different in Africa and Australia or, alternatively, a host animal is yet to be identified in Africa.

### 4.3. Other Countries

No study has reported *M. ulcerans* DNA or cases in non-human species in any country other than Australia. A study conducted by Heckert in 1997 at Chesapeake Bay, USA detected a new *Mycobacterium* species from wild striped bass [29]. This new isolate was closely related to *M. marinum*, *M. ulcerans*, and *M. tuberculosis*. Similarly, Sakaguchi and associates reported an atypical mycobacterial infection in an Indian flap-shelled turtle (*Lissemys punctata punctata)*, imported from India to Japan in an aquarium [26]. A PCR assay targeting the *rpoβ* gene revealed the isolate had 89–100% homology to *M. ulcerans* and *M. marinum*. Again, this study could not differentiate *M. ulcerans* from mycolactone-producing *M. marinum* (MPMM). Appleyard and Clark (2002) reported a new *Mycobacterial* species, namely ‘*Mycobacterium visibilis*’ from three cats initially suspected of having *M. ulcerans* infection [28].

## 5. Conclusions

Human cases of BU have been reported in more than 30 countries from Africa, America, Asia and Oceania. Since the implementation of PCR-based methods for the detection and identification of *M. ulcerans* from clinical and environmental samples, there has been a significant increase in overall knowledge of BU. There is no record of direct human-to-human transmission of *M. ulcerans*, unlike tuberculosis and leprosy. Australia is the only country where non-human cases of BU have been identified, with small mammals, especially possums and, to some extent, bandicoots, being implicated as potential reservoirs of *M. ulcerans*. Despite there having been several outbreaks in African countries, no non-human cases have been recorded so far and there is no evidence of any animal acting as a potential reservoir for this organism. None of the studies included in this review discussed strain variation of *M. ulcerans* in different geographical regions leading to an increase or decrease in susceptibility among animal or human population. Compared to other mycobacteria, such as *M. tuberculosis*, there is very little genetic diversity among isolates of *M. ulcerans*. Some variation among the strains of *M. ulcerans* from Africa, the Americas, Asia and the Western Pacific has been recorded; however, the linkage between these various strains and virulence in human or animal population has not been recognized so far. Remarkable differences in the type of mycolactone produced by *M. ulcerans* in different geographical location has been recorded. African strains produce more mycolactone variant A and B, whereas strains from Australia produce more mycolactone variant C. However, this variation has nothing to do with host susceptibility to *M. ulcerans*; rather, it determines cytopathogenecity and thus clinical presentation of disease.

This systematic review suggests the need for extensive laboratory and field research focusing on domestic animals and wildlife to elucidate their roles in BU-endemic countries.

## Figures and Tables

**Table 1:** Basic characteristics of selected studies on occurrence of *Mycobacterium ulcerans*.

Author and Year	Sample and Sample Size	Collection Year, Location and Setting	Detection Method, Result or *M. ulcerans* Positive Signal
Roltgen, Pluschke, Johnson, & Fyfe, 2017 [9]	102 environmental samples: 55 from soil/vegetation; 35 from insects or small insects pool and 12 from animal excreta	September 2013 Northern Queensland, Australia	RT-PCR IS 2404 positive: 1 soil specimen: 2 bandicoot faeces, one individual mosquito and 1 pool of 2 mosquitoes IS 2606 and KR (ketoreductase) positive: 2 bandicoot faeces and pool of two mosquitoes
Tobias et al., 2016 [23]	180 faecal specimens from dominant domestic animals (ovine, porcine, avian, reptiles, canine)	September 2013 4 BU-endemic and one non-endemic villages of Ghana, West Africa	RT-PCR IS 2404 positive: 2/86 ovine; 1/69 avian: 1/16 reptiles IS 2606 and KR: all negative
Tian, Niamke, Tissot-Dupont, &Drancourt, 2016 [24]	496 environmental samples: 100 from soil (endemic *n* = 50 and non-endemic *n* = 50); 200 from stagnant water (endemic *n* = 100 and non-endemic *n* = 100); 100 from plants (endemic *n* = 50 and non-endemic *n* = 50) and 96 animal faeces (*Thryonomys swinderianus* (agouti) stools) (endemic *n* = 48 and non-endemic *n* = 48)	June–October 2014 Ivory Coast, West Africa	RT-PCR 43 samples with at least one positive IS 2404 and KR Out of 43, only 10 positive for both IS2404 and KR, IS 2606 not performed: 7 water specimen; 2 *T. swinderianus* (agouti) faeces and one soil specimen
Carson et al., 2014 [5]	Fecal sample: 216 common ringtail possums and 6 common brushtail possums	Southeast Australia, State Victoria	RT-PCR targeting IS 2404, IS 2606 and KR 20 common ringtail possums and 4 common brushtail possums
O’Brien et al., 2014 [15]	69 possums (ringtail and brushtail) trapped at Point Lonsdale: Faecal samples: 57; blood samples: 63; buccal swab: 67; urine sample: 16; pouch swab: 15; cloacal swab: 20 69 fecal samples from 15 mountain brushtail possums	1998–2011 Victoria, Australia	RT-PCR targeting IS 2404, IS 2606 and KR Point Lonsdale: Positive: faecal sample: 12 (25%); blood sample: 0; buccal swab: 7 (16%); urine sample: 0; pouch swab: 3 (20%) Bellbird Creek: Positive: 4 mountain brushtail possums (27%)
C. O’Brien et al., 2013 [17]	Case report: two alpacas *(Vicugna pacos)* ulcerated tissue	Case 1: September 1997 Case 2: May 2011 Victoria, Australia	RT-PCR targeting IS 2404, IS 2606 and KR positive
Willson et al., 2013 [25]	587 fish representing 13 genera and 17 species and 351 amphibians representing 10 genera: external swab	2008–2009 Ghana, West Africa	RT-PCR targeting IS 2606 and KR not performed. Not confirmed
C. R. O’Brien et al., 2011 [18]	Case report: Case 1: 14 months old female kelpie Case 2: 3 years old female kelpie Case 3: 6 years old male whippet Case 4: 3 years old male koolie	2011 Victoria, Australia	RT-PCR targeting IS 2404, IS 2606 and KR All 4 dogs positive for *M. ulcerans*
Sakaguchi et al., 2011 [26]	Case report; Indian flap-shelled turtle, *Lissemys punctata punctata*	Imported from India to aquarium in Japan	PCR assays targeting the *rpoβ* gene: unable to differentiate *M. ulcerans* from mycolactone-producing *M. marinum* (MPMM)
Fyfe et al., 2010 [14]	589 fecal samples from ringtail possums and 250 samples from brushtail possums. Live trapping: 42 ringtail possums and 21 brushtail possums	2007–2009 Victoria, Australia	RT-PCR targeting IS 2404, IS 2606 and KR *M. ulcerans* DNA detected in 43% of ringtail possum and 29% of brushtail possum faecal samples. 38% ringtail possum have *M. ulcerans* lesion and/or positive faeces Lower in brushtail possums: 1 with *M. ulcerans* lesion and/or positive faeces and 4 with no lesions and low *M. ulcerans* DNA in faeces.
Durnez et al., 2010 [27]	565 small mammals: 326 rodents and 222 shrews	2006 Benin, West Africa	RT-PCR: No *M. ulcerans* specific DNA detected
Van Zyl et al., 2010 [16]	2 horses: Case report Case 1: 21-year-old quarterhorse-cross Case 2: 32-year-old standard bredgelding	Case 1: May 2006 Case 2: October 2006 Southeastern Australia	RT-PCR *M. ulcerans* specific DNA detected from both horses
Elsner et al., 2008 [19]	Cat: Case report 10-year-old castrated male domestic cat	2006 Victoria, Australia	RT-PCR *M. ulcerans* specific DNA detected
Appleyard & Clark, 2002 [28]	Case report: three cats Case 1: An 8-year-old spayed female shorthair Case 2: 6-year-old spayed female shorthair Case 3: 11-year-old domestic longhair cat	2002 North America	PCR Could not differentiate *M. ulcerans* from other *Mycobacterium* spp. (a new *Mycobacterial* spp. namely ‘*Mycobacterium visibilis*’ suggested)
Heckert, Elankumaran, Milani, &Baya, 2001 [29]	60 wild striped bass: Swab from external ulcerative dermatitis and granulomatous-like lesions in the internal organs	1997 Chesapeake Bay, USA	PCR No *M. ulcerans* specific DNA detected (a new mycobacterial spp. suggested)
Mitchell, McOrist, &Bilney, 1987 [13]	36 male and 51 female adult koalas captured	1980–1985 Raymond Island, southeastern Australia	Pathological and bacteriological examination 18 out of 87 captured koalas had skin wound 11 koalas were found positive for *M. ulcerans*
McOrist, Jerrett, Anderson, & Hayman, 1985 [12]	Case study: 2 koalas: one male and one female Ulcerated tissue	1982 Raymond Island, southeastern Australia	Pathological and bacteriological examination Both koalas suggested positive for *M. ulcerans*

## References

[1] Alsop D.G. (1972). The Bairnsdale ulcer. Aust. N. Z. J. Surg..

[2] MacCallum P.T.J.C., Tolhurst J.C., Buckle G., Sissons H.A. (1948). A new mycobacterial infection in man. J. Pathol. Bacteriol..

[3] Clancey J., Dodge R., Lunn H.F. (1920). Study of a *Mycobacterium* causing skin ulceration in Uganda. Ann. Soc. Belg. Med. Trop..

[4] Johnson P.D., Azuolas J., Lavender C.J., Wishart E., Stinear T.P., Hayman J.A., Brown L., Jenkin G.A., Fyfe J.A. (2007). *Mycobacterium ulcerans* in mosquitoes captured during outbreak of Buruli ulcer, southeastern Australia. Emerg. Infect. Dis..

[5] Carson C., Lavender C.J., Handasyde K.A., O’Brien C.R., Hewitt N., Johnson P.D., Fyfe J.A. (2014). Potential wildlife sentinels for monitoring the endemic spread of human Buruli ulcer in south-east Australia. PLoS Negl. Trop. Dis..

[6] World Health Organization (2018). Neglected Tropical Diseases. http://www.who.int/neglected_diseases/diseases/en/.

[7] World Health Organization (2014). Distribution of Buruli Ulcer, Worldwide 2014.

[8] Steffen C.M., Smith M., McBride W.J. (2010). *Mycobacterium ulcerans* infection in North Queensland: The ‘Daintree ulcer’. ANZ J. Surg..

[9] Röltgen K., Pluschke G., Johnson P.D., Fyfe J. (2017). *Mycobacterium ulcerans* DNA in bandicoot excreta in Buruli ulcer-endemic area, northern Queensland, Australia. Emerg. Infect. Dis..

[10] Francis G., Whitby M., Woods M. (2006). *Mycobacterium ulcerans* infection: A rediscovered focus in the Capricorn Coast region of central Queensland. Med. J. Aust..

[11] Radford A.J. (1975). *Mycobacterium ulcerans* in Australia. Aust. N. Z. J. Med..

[12] McOrist S., Jerrett I.V., Anderson M., Hayman J. (1985). Cutaneous and respiratory tract infection with *Mycobacterium ulcerans* in two koalas (*Phascolarctos cinereus*). J. Wildl. Dis..

[13] Mitchell P.J., McOrist S., Bilney R. (1987). Epidemiology of *Mycobacterium ulcerans* infection in koalas (*Phascolarctos cinereus*) on Raymond Island, southeastern Australia. J. Wildl. Dis..

[14] Fyfe J.A., Lavender C.J., Handasyde K.A., Legione A.R., O’Brien C.R., Stinear T.P., Pidot S.J., Seemann T., Benbow M.E., Wallace J.R. (2010). A major role for mammals in the ecology of *Mycobacterium ulcerans*. PLoS Negl. Trop. Dis..

[15] O’Brien C.R., Handasyde K.A., Hibble J., Lavender C.J., Legione A.R., McCowan C., Globan M., Mitchell A.T., McCracken H.E., Johnson P.D. (2014). Clinical, microbiological and pathological findings of *Mycobacterium ulcerans* infection in three Australian possum species. PLoS Negl. Trop. Dis..

[16] van Zyl A., Daniel J., Wayne J., McCowan C., Malik R., Jelfs P., Lavender C.J., Fyfe J.A. (2010). *Mycobacterium ulcerans* infections in two horses in south-eastern Australia. Aust. Vet. J..

[17] O’Brien C., Kuseff G., McMillan E., McCowan C., Lavender C., Globan M., Jerrett I., Oppedisano F., Johnson P., Fyfe J. (2013). *Mycobacterium ulcerans* infection in two alpacas. Aust. Vet. J..

[18] O’Brien C.R., McMillan E., Harris O., O’Brien D.P., Lavender C.J., Globan M., Legione A.R., Fyfe J.A. (2011). Localised *Mycobacterium ulcerans* infection in four dogs. Aust. Vet. J..

[19] Elsner L., Wayne J., O’Brien C.R., McCowan C., Malik R., Hayman J.A., Globan M., Lavender C.J., Fyfe J.A. (2008). Localised *Mycobacterium ulcerans* infection in a cat in Australia. J. Feline Med. Surg..

[20] Moher D., Liberati A., Tetzlaff J., Altman D.G., Prisma Group (2010). Preferred reporting items for systematic reviews and meta-analyses: The PRISMA statement. Int. J. Surg..

[21] PROSPERO Registered Study Protocol. https://www.crd.york.ac.uk/prospero/display_record.php?RecordID=85484.

[22] Fyfe J.A., Lavender C.J., Johnson P.D., Globan M., Sievers A., Azuolas J., Stinear T.P. (2007). Development and application of two multiplex real-time PCR assays for the detection of *Mycobacterium ulcerans* in clinical and environmental samples. Appl. Environ. Microbiol..

[23] Tobias N.J., Ammisah N.A., Ahortor E.K., Wallace J.R., Ablordey A., Stinear T.P. (2016). Snapshot faecal survey of domestic animals in rural Ghana for *Mycobacterium ulcerans*. PeerJ.

[24] Tian R.B., Niamké S., Tissot-Dupont H., Drancourt M. (2016). Detection of *Mycobacterium ulcerans* DNA in the environment, Ivory Coast. PLoS ONE.

[25] Willson S.J., Kaufman M.G., Merritt R.W., Williamson H.R., Malakauskas D.M., Benbow M.E. (2013). Fish and amphibians as potential reservoirs of *Mycobacterium ulcerans*, the causative agent of Buruli ulcer disease. Infect. Ecol. Epidemiol..

[26] Sakaguchi K., Iima H., Hirayama K., Okamoto M., Matsuda K., Miyasho T., Kasamatsu M., Hasegawa K., Taniyama H. (2011). *Mycobacterium ulcerans* infection in an Indian flap-shelled turtle (*Lissemys punctata punctata*). J. Vet. Med. Sci..

[27] Durnez L., Suykerbuyk P., Nicolas V., Barriere P., Verheyen E., Johnson C.R., Leirs H., Portaels F. (2010). Terrestrial small mammals as reservoirs of *Mycobacterium ulcerans* in Benin. Appl. Environ. Microbiol..

[28] Appleyard G.D., Clark E.G. (2002). Histologic and genotypic characterization of a novel *Mycobacterium* species found in three cats. J. Clin. Microbiol..

[29] Heckert R.A., Elankumaran S., Milani A., Baya A. (2001). Detection of a new *Mycobacterium* species in wild striped bass in the Chesapeake Bay. J. Clin. Microbiol..

[30] Ross B.C., Marino L., Oppedisano F., Edwards R., Robins-Browne R.M., Johnson P.D. (1997). Development of a PCR assay for rapid diagnosis of *Mycobacterium ulcerans* infection. J. Clin. Microbiol..

[31] Portaels F., Hibble J. (2001). *Mycobacterium ulcerans* in wild animals. Rev. Sci. Technol..

